# Buspirone combats cyclophosphamide-provoked hepatotoxicity in rats via activation of AMPK/Nrf2/HO-1 and suppression of NF-κB p65 /NLRP3 inflammasome pathways

**DOI:** 10.1007/s00210-025-04718-3

**Published:** 2025-11-03

**Authors:** Marwa M. Khalaf, Ehab E. Sharata, Waleed A. I. Khallaf, Mina Ezzat Attya, Amira M. Abo-Youssef, Ramadan A. M. Hemeida, Remon Roshdy Rofaeil

**Affiliations:** 1 Department of Pharmacology & Toxicology, Faculty of Pharmacy, Beni-Suef University, Beni-Suef, 62514 Egypt; 2 Department of Pharmacology & Toxicology, Faculty of Pharmacy, Deraya University, Minia, 61111 Egypt; 3 Department of Pathology, Faculty of Medicine, Minia University, Minia, 61519 Egypt; 4 Department of Medical Pharmacology, Faculty of Medicine, Minia University, Minia, 61519 Egypt

**Keywords:** Cyclophosphamide, Hepatic injury, Buspirone, NLRP3, Nrf2, α-Klotho

## Abstract

This study aims to evaluate the protective effect of buspirone (BUS) against liver damage caused by cyclophosphamide (CPA) by focusing on the α-klotho/Nrf2/HO-1 and AMPK/NF-κB p65/NLRP3/caspase-1 signaling cascades. The possible damage that CPA might produce was evaluated using histological examination in conjunction with serum AST, ALT, and direct bilirubin. GSH and MDA levels were measured using a colorimetric technique. TNF-α, IL-1β, and IL-18 levels, as well as hepatic caspase-1, hepatic p-AMPK, and serum α-klotho, were measured using the ELISA technique. Using an immunohistochemistry method, the Nrf2 and caspase-3 expression in the liver tissue was investigated. The expression of HO-1 mRNA was assessed by means of RT-qPCR. The expression levels of NF-κB p65 and NLRP3 were assessed by western blotting. BUS, in a dose-dependent manner, attenuated CPA-induced hepatotoxicity by reducing the elevated serum AST, ALT, and direct bilirubin and alleviating the histopathological aberrations. Additionally, it raised GSH levels and decreased MDA levels. In addition, it reduced levels of inflammatory markers and caspase-3 expression. BUS also increased p-AMPK and α-klotho protein levels and stimulated the production of Nrf2 and HO-1. Additionally, it reduced pyroptosis by downregulating NLRP3 and caspase-1 expression levels. BUS attenuated NF-κB p65/NLRP3 inflammasome and caspase-3-mediated apoptotic activity and enhanced Nrf2/HO-1 activity, therefore mitigating the liver impairment provoked by CPA.

**Supplementary Information:**

The online version contains supplementary material available at 10.1007/s00210-025-04718-3.

## Introduction

Cancer continues to be a prominent issue in global health and is one of the primary contributors to mortality on a worldwide scale. Approximately 19.3 million new cases of cancer and 10 million deaths were attributed to cancer in 2020, according to the World Health Organization (Sung et al. 2021). Chemotherapy is still the mainstay of cancer treatment (Anand et al. 2023), despite the advent of novel medicines, including hormone therapy or immunotherapy, with superior efficacy and a drug-specific adverse profile (Porras et al. 2023). Chemotherapeutic drugs are linked to a variety of negative effects, including hepatotoxicity, and account for 10% of all medicines that cause liver injury (Björnsson 2016). Chemotherapy-related liver dysfunction is more prevalent with methotrexate, cisplatin, and cyclophosphamide (Dass 2020).

One common and effective alkylating medicine is cyclophosphamide (CPA), which is used to treat many malignant diseases, such as lymphomas and breast cancer (Abu-Risha et al. 2022). The immunomodulatory features of CPA make it a crucial strategy for addressing immunological disorders such as systemic lupus erythematosus, rheumatoid arthritis, and multiple sclerosis (Abd Elmaaboud et al. 2024). Despite the fact that CPA has a wide variety of therapeutic applications, its effectiveness is limited due to certain adverse outcomes, such as hepatotoxicity, which have been documented in various clinical situations (Zhu et al. 2015; Ming et al. 2019; Subramaniam et al. 2013). Therefore, finding new and effective hepatoprotective medicines is crucial to increase its therapeutic utility.

CPA is a prodrug that is metabolically activated by the liver, resulting in the production of highly hazardous metabolites such as acrolein and phosphoramide mustard (Li et al. 2010). CPA’s antineoplastic action has been correlated with phosphoramide mustard, whereas its noxious impact is attributed to acrolein. The liver simultaneously mitigates the detrimental effects of these hazardous substances through its inherent detoxification system (Zhu et al. 2015; Saleh et al. 2024). Acrolein decreases levels of glutathione (GSH) and superoxide dismutase (SOD) in the liver while it undergoes detoxification. The outcome is an increase in the production of ROS, which can cause oxidative stress and damage to the liver (Saleh et al. 2024; Zhang et al. 2021). Moreover, nuclear factor kappa B (NF-κB) activity is upregulated due to a buildup of ROS (El-Beheiry et al. 2023). Both ROS and NF-κB facilitate the activation of the nucleotide-binding oligomerization domain-like receptor family pyrin domain-containing 3 (NLRP3) inflammasome (Wei et al. 2021; Matouk et al. 2022). Upon activation of the NLRP3 inflammasome, proinflammatory cytokines, including interleukin 1β (IL-1β) and interleukin 18 (IL-18), are processed by caspase 1 into their active forms (Khallaf et al. 2023). Several recent investigations have found that CPA-induced liver damage is associated with NLRP3 inflammasome activation (Ma et al. 2021; Mostafa et al. 2022; Mansour et al. 2017a).

Nuclear factor erythroid 2-related factor 2 (Nrf2) is a vital sensor that shields cells from oxidative damage brought on by xenobiotics (Kensler et al. 2007). Nrf2 subsequently activates the protective antioxidant enzyme system, leading to a reduction in inflammation, oxidative stress, and hepatocellular injury (Al-Amarat et al. 2022). Multiple investigations have shown that CPA-induced liver damage is significantly influenced by hepatic Nrf2/HO-1 downregulation (Sherif 2018; Mahmoud et al. 2017). Furthermore, a lack of Nrf2 enhances the activation of NF-κB, resulting in aggravated stimulation of the NLRP3 inflammasome and caspase-3-mediated cell death (El-Agamy et al. 2018; Hou et al. 2018).

Numerous critical physiological processes, such as energy consumption, anti-inflammatory reactions, and the regulation of oxidative stress, depend on adenosine monophosphate-activated protein kinase (AMPK) (El-Dessouki et al. 2024). The function of AMPK in preventing oxidative stress and liver damage brought on by chemotherapy has been the subject of an increasing number of studies recently (Bokhary et al. 2022; Xu et al. 2022). Stimulation of AMPK has been shown to increase Nrf2/HO-1 expression (Fischhuber et al. 2020) and downregulate the NLRP3 inflammasome via modulation of NF-κB (Abd El-Fattah et al. 2022).

α-Klotho, a gene linked to anti-aging, has recently attracted a lot of attention due to its multipurpose characteristics (Chi et al. 2023). The α-klotho in circulation counteracts oxidative stress, slows down the ageing process, and prevents cell death (Kim et al. 2013). New research has revealed a connection between circulating α-klotho deficiency, liver fibrosis, and hepatic dysfunction in non-alcoholic fatty liver disease (Chi et al. 2023; Liu et al. 2022). However, studies examining its specific alleviating effects on hepatic dysfunction caused by CPA are limited. In addition, new research has shown that α-klotho amplification suppresses NLRP3-mediated pyroptosis (Li et al. 2019), and oxidative stress (Oh et al. 2015) while stimulating Nrf2 signaling cascade (Xing et al. 2021).

Buspirone (BUS) is a medication that is frequently prescribed to patients suffering from depression and anxiety (Loane And Politis 2012). It accomplishes its purpose by partly triggering the 5-HT_1A_ receptor. Additionally, the medicine blocks the dopamine D_2_ receptor family. The release of 5-HT at nerve terminals is restricted, and 5-HT neuronal activity is inhibited when presynaptic 5-HT_1A_ receptors are activated (Abdel-Salam et al. 2017). Cancer patients using chemotherapy drugs, such as CPA, sometimes experienced nausea, vomiting, and difficulty breathing; BUS was used as an adjuvant to alleviate these side effects (Alfieri And Cubeddu 1995; Wolff And Leander 1997; Peoples et al. 2016). Apart from its ability to prevent vomiting, BUS also improves stomach accommodation and alleviates gastroparesis (Parkman et al. 2023; Sayuk 2023). Gastroparesis can occasionally occur in cancer patients due to real cancer itself or chemotherapy-induced neuropathy (Donthireddy et al. 2007; Kelly et al. 2014).

The evidence supporting this study shows that BUS protected against methotrexate-induced hippocampal damage, activated the Nrf2/HO-1 signaling pathway, and suppressed the NLRP3 inflammasome (Althagafy et al. 2023). Additionally, it mitigated colon inflammation through the Toll-like receptor 4 (TLR4)/NF-κB pathway in TNBS-induced acute colitis (Rashidian et al. 2022). In hypertensive rats, BUS also increased AMPK, which aided in weight loss and the correction of metabolic abnormalities (Lee et al. 2023). Furthermore, earlier experimental research has demonstrated that BUS can lessen the liver damage brought on by methylphenidate and carbon tetrachloride (Abdel-Salam et al. 2017; Alam And Ikram 2018). These factors led us to hypothesize that BUS may be a useful treatment to lessen the hepatotoxicity caused by CPA. Given the significance of the prior findings, the present investigation was conducted to elucidate the potential mechanism of action of BUS and its protective efficacy against CPA-induced hepatotoxicity in rats.

## Materials and methods

### Drugs and chemical agents

Germany’s Baxter Company was the prospective purchaser of CPA. SmithKline Beecham was the one that supplied BUS.

### Housing of animals

Male Wistar albino rats ranging in age from 6 to 9 weeks and weighing 180 to 210 g were supplied by the National Research Center (Giza, Egypt) for the study. The rats received limitless amounts of water and food pellets for the week before the experiment started to help them adjust to the lab setting. They were housed in a controlled setting that consistently provided 12 h of light, 25 ± 2 °C of temperature, and 45 ± 5% humidity. The research procedure was approved by the Deraya University Institutional Animal Care and Use Committee (DCSR-08024–15), Deraya University, Egypt, in accordance with NIH requirements. The current research also complies with ARRIVE guidelines.

### Experimental design

**Fig. 1:**
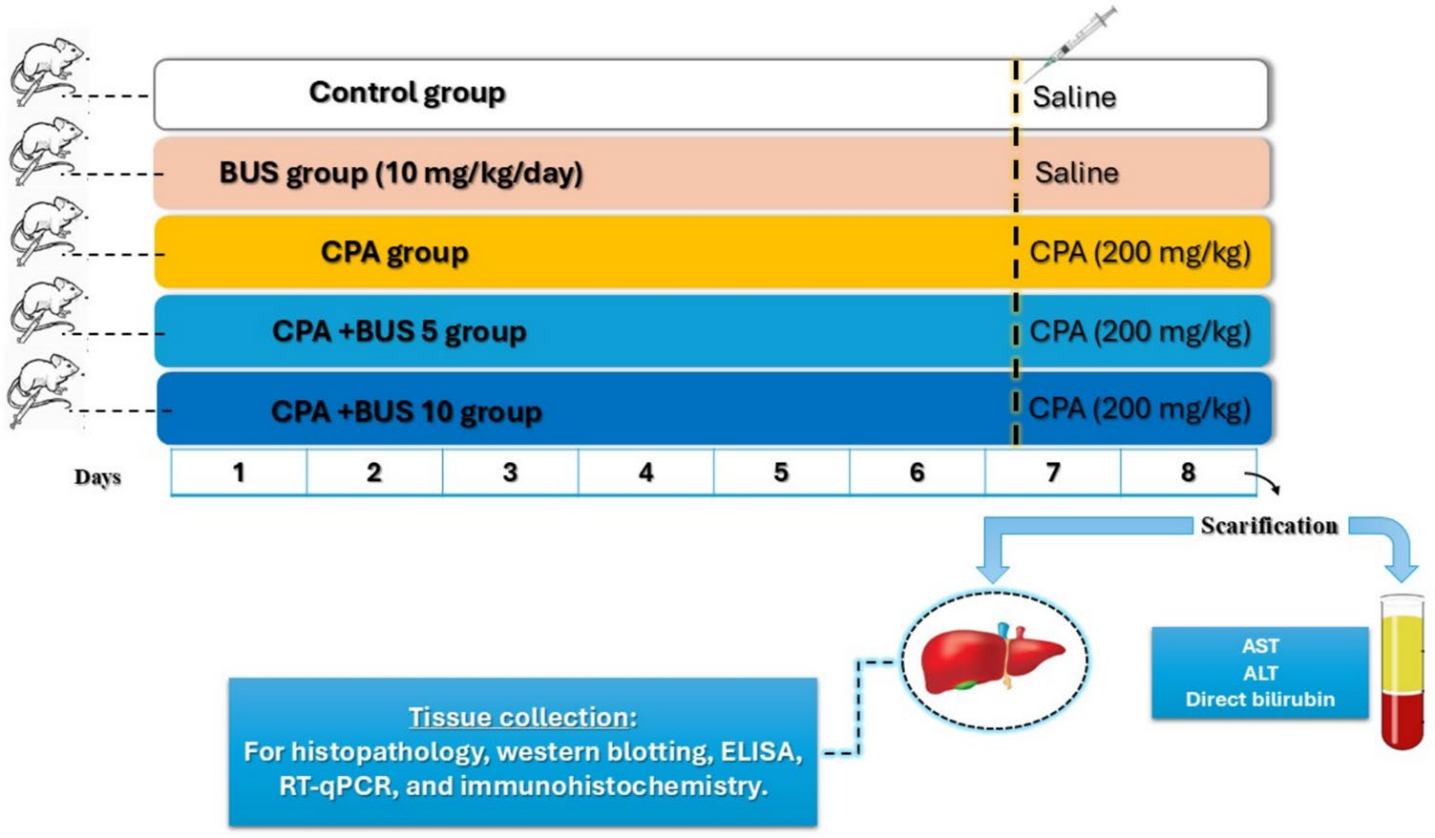
Graph illustrating the study’s methodology and treatment plan. Male rats were randomly allocated into five groups: Group I served as control receiving saline for 7 days; Group II received BUS alone at 10 mg/kg/day orally for 7 days; Group III received a single i.p. dose of CPA 200 mg/kg on day 7; Groups IV and V were co-treated with BUS at 5 or 10 mg/kg/day orally for 7 days plus CPA 200 mg/kg i.p. on day 7. All rats were euthanized 24-h post-CPA injection. Liver tissues and blood samples were collected for biochemical, molecular, and histological analyses. BUS: buspirone, CPA: cyclophosphamide, AST: aspartate transaminase, and ALT: alanine transaminase

As shown in Fig. 1, the animals were randomly divided into five groups, with ten animals in each group. The animals were grouped as follows:

I. Control group: the rats were administered normal saline solution for 7 days.
II. BUS group: BUS dissolved in normal saline was administered at a dose of 10 mg/kg/day orally for 7 days.
III. CPA group: one dose of CPA (200 mg/kg; i.p.) was given to the animals on the 7th day of the experiment.
IV. CPA + BUS 5 group: For 7 days, animals were given BUS at a dosage of 5 mg/kg/day orally, along with a single dose of CPA at a dose of 200 mg/kg given intraperitoneally on day 7.
V. CPA + BUS 10 group: For 7 days, animals were given BUS at a dosage of 10 mg/kg/day orally, along with a single dose of CPA at a dose of 200 mg/kg given intraperitoneally on day 7.

The BUS doses were established in light of recent studies that have shown its pharmacological capacity to reduce inflammation and oxidative damage (Abdel-Salam et al. 2017; Rashidian et al. 2022). The utilized CPA dosage has been documented to cause hepatotoxicity in many experiments (Saleh et al. 2024; Shokrzadeh et al. 2014). The administration of BUS continued daily for 6 days before the administration of CPA. CPA was administered 4 h after BUS administration on the 7th day.

### Tissue isolation

Rats were sacrificed 24-h post-CPA injection. Rats were received a 1.3-g/kg intraperitoneal urethane injection after overnight fasting (Rofaeil et al. 2025), then euthanized by cervical dislocation to collect blood samples and liver tissue. Blood was drawn from the abdominal aorta of rats and centrifuged for 15 min at 4000 g to conduct a liver function panel (Sharata et al. 2025a). The liver tissues were removed and then rinsed with saline. After dividing the liver tissues into parts, the first part (the left lateral lobe) was promptly frozen at − 80 °C to facilitate Western blot analysis. Histological and immunohistochemical examinations required fixing the second part (the right and caudate lobes) in 10% formalin. For further homogenization and assessment of oxidative stress and inflammatory indicators, the residual portion (the median lobe and remaining liver tissue) was stored at − 80 °C.

### Biochemical assessment

#### Estimation of hepatic function parameters

Quantitation of ALT, AST, and direct bilirubin levels was done as part of the liver function panel assessment by adherently following the manufacturer’s directions; colorimetric kits were used (Bio Diagnostic, Egypt).

#### Evaluation of oxidative stress indicators

Following the manufacturer’s instructions, the MDA and GSH contents in the liver were assessed by detection kits (Bio Diagnostic, Egypt).

#### ELISA measurements

According to the instructions provided by the manufacturer, ELISA kits from Cloud-Clone Corp, USA, were used to detect hepatic TNF-α (SEA133Ra), IL-18 (SEA064Ra), and IL-1β (SEA563Ra). Hepatic caspase 1 was evaluated utilizing an ELISA kit (E-EL-R0371, Elabscience, USA). An ELISA kit (E1206Ra, BT LAB, China) was used to measure the serum level of α-klotho. Hepatic HO-1 was measured using an ELISA kit (E-EL-R0488, Elabscience, USA).

### Western blotting

The western blotting analysis was performed using the liver homogenate following the previously mentioned procedures (Sharata et al. 2025a). The p-AMPK alpha 1 (phospho T183) (ab133448, Abcam, UK), AMPK alpha 1 antibody (ab32047, Abcam, UK), NF-kB p65 antibody (ab32536, Abcam, UK), NLRP3 antibody (ab263899, Abcam, UK), and β-actin antibody (Santa Cruz Biotechnology, Santa Cruz) were utilized for primary blotting. The following stage was the use of a secondary antibody, a goat anti-rabbit IgG, in combination with horseradish peroxidase (7074S, Cell Signaling Technology, USA).

### Immunohistochemistry

After being dewaxed, 4-μm-thick paraffin sections were submerged in a citrate buffer solution (0.05 M, pH 6.8) to extract the antigen. After that, these parts were treated with a protein block and 0.3% H_2_O_2_. The next step was to apply Nrf2 antibodies (Servicebio, China) and caspase-3 antibodies (Chongqing Biospes Co., China) to the samples. A 30-min incubation with secondary antibodies conjugated with HRP was performed on the samples after a complete washing in phosphate-buffered saline. The DAB kit was used for visualization before Mayer’s hematoxylin staining of the slides. When the slides were viewed under a light microscope, the positive level in the hepatic tissues was graded using the following system: There is no immune expression at all when the score is 0, mild expression when it is 1, moderate expression when it is 2, and vigorous expression when it is 3 (Mohyeldin et al. 2024).

### RT-qPCR for the HO-1 gene

The RT-qPCR analysis was performed using the RNA that was isolated from hepatic tissue using the previously described procedures (El-Beheiry et al. 2023). The primer sequences for the target genes are listed in Table 1. Gene expression was measured using the ^2−ΔΔ Ct^ method using GAPDH as an internal reference.

**Table 1:** The sequence of the primer

Genes	Primer	
***HO-1***	F	5’-GACAGAAGAGGCTAAGACCGC-3’
	R	5’-TGGAGGAGCGGTGTCTGG-3’
***GAPDH***	F	5’- CTCTCTGCTCCTCCCTGTTC -3’
	R	5’- CGACATACTCAGCACCAGCA -3’

### Histological examination

Quickly, we extracted the livers from each animal and preserved them in a 10% neutral-buffered formalin solution. Before examination under a light microscope (Olympus, U.TV0.5XC-3), the samples underwent processing and staining following conventional hematoxylin and eosin protocols (Bancroft and Layton 2013). Characteristics such as hepatocyte necrosis, apoptosis, inflammation, central venous congestion, dilated congested sinusoids, steatosis, and diplocyte presence were employed in the semi-quantitative scoring technique throughout the evaluation phase. Tissue damage was quantified using the scores, which ranged from 0 (no damage), 1 (mild damage), 2 (moderate damage), and 3 (severe) for each parameter. The results were thereafter shown as an aggregate of the individual grade points (Mohyeldin et al. 2023).

### Statistical analysis

The mean ± SD was used to present the data. To find statistically significant changes between groups, this study employed GraphPad Prism (version 7, USA) and ran a one-way ANOVA, followed by a Tukey test for parametric data and a Kruskal–Wallis test, followed by Dunn’s multiple comparison test for non-parametric data. a probability value less than 0.05 was deemed significant.

## Results

### BUS mitigated CPA-induced changes in liver function panels

Table 2 illustrates the data collected to assess the preventative impact of BUS on CPA-induced hepatic damage. Our findings revealed that the control and BUS-monotherapy groups exhibited identical normal levels for ALT, AST, and direct bilirubin. Conversely, administration of CPA led to a significant elevation (*p* < 0.001) in ALT, AST, and direct bilirubin levels, as contrasted with the control group. Fortunately, the groups that received a combination of CPA + BUS at doses of 5 mg/kg and 10 mg/kg exhibited a notable dose-dependent decrease (*p* < 0.001) in ALT, AST, and direct bilirubin levels, as compared with rats that were just given CPA.

**Table 2:** Effect of BUS on the liver function panel (ALT, AST, and direct bilirubin)

Group	ALT (U/L)	AST (U/L)	Direct bilirubin (mg/dL)
Control	55.86 ± 5.95	103.30 ± 15.90	0.77 ± 0.15
BUS 10	54.91 ± 6.49	92.41 ± 14.92	0.80 ± 0.21
CPA	203.40 ± 11.90^###^	302.90 ± 13.80^###^	4.90 ± 0.34^###^
CPA + BUS 5	144.40 ± 10.72^###, ***^	198.10 ± 14.58^###, ***^	3.84 ± 0.51^###, ***^
CPA + BUS 10	89.52 ± 8.24^###, ***, $$$^	136.30 ± 15.11^###, ***,^ ^$$$^	3.21 ± 0.37^###, ***,^ ^$^

The mean ± SD is utilized to express the data (*n* = 6). The significance of differences was evaluated using one-way ANOVA, followed by the Tukey–Kramer post hoc test, where ^**###**^*p* < 0.001, relative to the control group, ***p < 0.001, relative to the CPA group, ^$^*p* < 0.05, relative to CPA + BUS 5 group, and ^$$$^*p* < 0.001, relative to CPA + BUS 5 group. *BUS* buspirone, *CPA* cyclophosphamide, *ALT* alanine transaminase, and *AST* aspartate transaminase

### BUS restored antioxidant balance

**Fig. 2:**
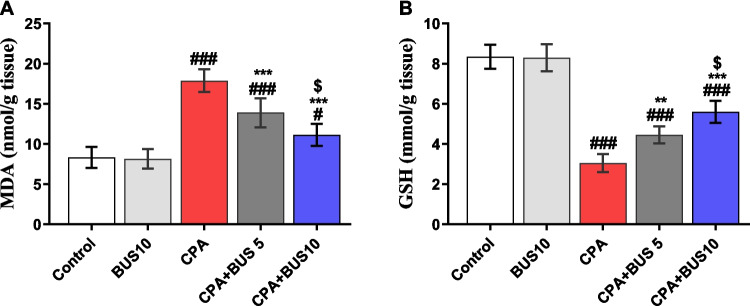
The influence of BUS on hepatic MDA (**A**) and GSH (**B**) levels. The mean ± SD is utilized to express the data (*n* = 6). The significance of differences was evaluated using one-way ANOVA, followed by the Tukey–Kramer post hoc test, where ^**#**^*p* < 0.05, relative to the control group, ^**###**^*p* < 0.001, relative to the control group, **p < 0.01, relative to the CPA group, ***p < 0.001, relative to the CPA group, and ^$^*p* < 0.05, relative to CPA + BUS 5 group. BUS: buspirone, CPA: cyclophosphamide, MDA: malondialdehyde, and GSH: reduced glutathione

Hepatic antioxidant capacity and lipid peroxidation rate were assessed by measuring GSH and MDA levels in liver homogenates. Compared to the control group, the MDA content showed a significant increase (*p* < 0.001) after CPA administration (Fig. 2A). Surprisingly, the combination of CPA + BUS effectively reduced (*p* < 0.001) the MDA levels to lower values. In addition, MDA analysis showed a substantial reduction (*p* < 0.05) when comparing the CPA + BUS 10 group to the CPA + BUS 5 group. Additionally, we assessed the hepatic GSH content as a measure of the antioxidant capability of the liver to identify the extent to which BUS restored redox hemostasis. In comparison to the control group, CPA treatment resulted in a significant reduction (*p* < 0.001) in hepatic GSH content. On the other hand, as shown in Fig. 2B, the GSH levels in the CPA + BUS 5 and 10 groups demonstrated a significant increase (*p* < 0.01, *p* < 0.001, respectively) in comparison to the CPA group, and this increase was dose-dependent.

### BUS attenuated hepatic inflammation

We assessed the impact of BUS on the inflammatory response triggered by CPA by evaluating the protein expression of inflammatory cytokines using the ELISA technique. Comparing the hepatic tissue of CPA-intoxicated rats to the control group, we observed an alarming rise (*p* < 0.001) in the levels of TNF-α, IL-18, and IL-1β. On the flip side, pretreatment with BUS showed anti-inflammatory effects, as evidenced by a dose-dependent, notable decrease (*p* < 0.001) in hepatic TNF-α, IL-18, and IL-1β compared to the CPA-intoxicated group, as depicted in Table 3.

**Table 3:** Effect of BUS on hepatic inflammatory parameters (TNF-α, IL-18, and IL-1β)

Group	Hepatic TNF-α (pg/g tissue)	Hepatic IL-18 (pg/g tissue)	Hepatic IL-1β (pg/g tissue)
Control	221.40 ± 15.77	183.80 ± 16.97	250.10 ± 34.21
BUS 10	218.00 ± 14.79	178.20 ± 17.26	259.80 ± 48.88
CPA	455.50 ± 25.39^###^	363.50 ± 26.92^###^	588.10 ± 68.72^###^
CPA + BUS 5	320.20 ± 26.27^###, ***^	293.40 ± 17.11^###, ***^	450.70 ± 56.51^###, ***^
CPA + BUS 10	258.90 ± 19.45^#, ***, $$$^	213.00 ± 17.02^***, $$$^	357.90 ± 38.13^##, ***, $^

The mean ± SD is utilized to express the data (*n* = 6). The significance of differences was evaluated using one-way ANOVA, followed by the Tukey–Kramer post hoc test, where ^**#**^*p* < 0.05, relative to the control group, ^**##**^*p* < 0.01, relative to the control group, ^**###**^*p* < 0.001, relative to the control group, ***p < 0.001, relative to the CPA group, ^$^*p* < 0.05, relative to CPA + BUS 5 group, and ^$$$^*p* < 0.001, relative to CPA + BUS 5 group. *BUS* buspirone, *CPA* cyclophosphamide, *TNF-α* tumor necrosis factor-alpha, *IL-18* interleukin-18, and *IL-1β* interleukin-1β

### BUS upregulated hepatic p-AMPK and attenuated hepatic pyroptosis

**Fig. 3:**
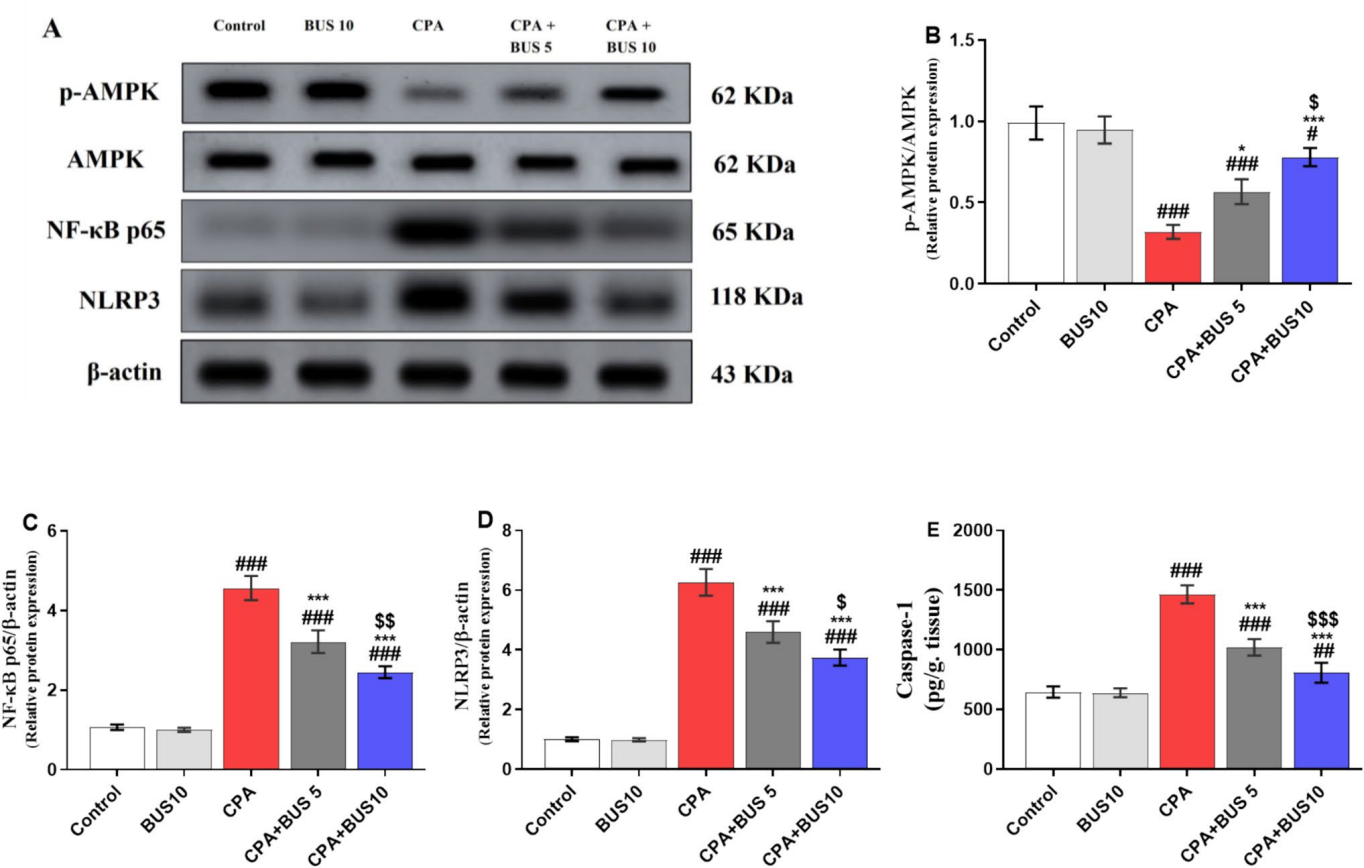
Immunoblotting panel of hepatic p-AMPK, NF-κB p65, and NLRP3, (**A**), densitometric quantification of hepatic p-AMPK (**B**), NF-κB p65 (**C**), NLRP3 (**D**), and ELISA assay of caspase-1 (**E**). The mean ± SD is used to express the data (*n* = 3 for Western and *n* = 6 for ELISA). The significance of differences was evaluated using one-way ANOVA, followed by the Tukey–Kramer post hoc test, where^#^*p* < 0.05, relative to the control group, ^#^*p* < 0.01, relative to the control group, ^###^*p* < 0.001, relative to the control group, *p < 0.05, relative to the CPA group, ***p < 0.001, relative to the CPA group, ^$^*p* < 0.05, relative to CPA + BUS 5 group, ^$$^*p* < 0.01, relative to CPA + BUS 5 group and.^$$$^*p* < 0.001, relative to CPA + BUS 5 group. BUS: buspirone, CPA: cyclophosphamide, NF-κB p65: nuclear factor-kappa B p65, and NLRP3: NOD-like receptor family pyrin domain containing 3The significance of differences was evaluated using the Kruskal–Wallis test, followed by Dunn’s multiple comparison test (*n* = 6), where ^>##^*p* < 0.01, relative to the control group,^###^*p* < 0.001, relative to the control group, *p < 0.05, relative to the CPA group, ***p < 0.001, relative to the CPA group, and ^$$^*p* < 0.01, relative to the CPA + BUS 5 group. *BUS* buspirone, *CPA* cyclophosphamide.

Figure 3 showed a substantial decrease (*p* < 0.001) in hepatic p-AMPK in the CPA group relative to the control group. Surprisingly, the expression levels of hepatic p-AMPK demonstrated a significant increase (*p* < 0.05, *p* < 0.001, respectively) in the CPA + BUS 5 and CPA + BUS 10 groups compared to the CPA group. In addition, when comparing CPA + BUS 10 to the CPA + BUS 5 group, there was a notable increase (*p* < 0.05) in p-AMPK. Additionally, to assess the potential antipyroptotic effect of BUS. Firstly, we used the Western technique to measure the NF-κB p65 and NLRP3 protein expression levels. Figure 3 showed that CPA elicited a significant increase (*p* < 0.001) in both NF-κB p65 and NLRP3 protein levels in contrast to the control group. On the other hand, pretreatment with BUS resulted in a significant dose-dependent decline (*p* < 0.001) in hepatic NF-κB p65 and NLRP3 content compared to the CPA group. Furthermore, CPA + BUS 10 showed a substantial decrease (*p* < 0.01) in both NF-κB p65 and NLRP3 protein levels compared to the CPA + BUS 5 group. Secondly, we assessed caspase-1 protein expression using the ELISA technique. Figure 3E demonstrated that CPA markedly enhanced (*p* < 0.001) the expression of caspase-1 as compared to the control group. In contrast to the CPA group, the BUS-treated rats displayed a significant decrease (*p* < 0.001) in the expression of hepatic caspase-1 in a dose-dependent manner.

### BUS boosted α-klotho serum levels

**Fig. 4:**
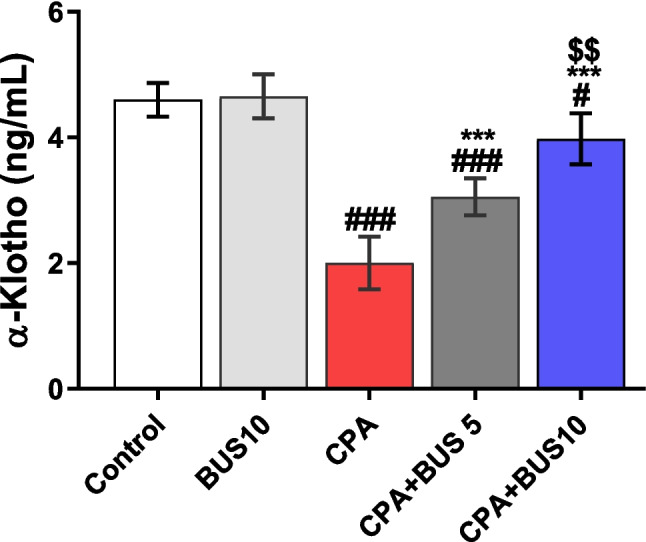
The influence of BUS on serum α-klotho. The mean ± SD is utilized to express the data (*n* = 6). The significance of differences was evaluated using one-way ANOVA, followed by the Tukey–Kramer post hoc test, where ^**#**^*p* < 0.05, relative to the control group, ^**###**^*p* < 0.001, relative to the control group, ***p < 0.001, relative to the CPA group, and ^$$^*p* < 0.01, relative to the CPA + BUS 5 group. BUS: buspirone, and CPA: cyclophosphamide

A significant decrease (*p* < 0.001) in serum α-klotho levels was observed in the group that received CPA, as compared to the control group. Contrary, both the CPA + BUS 5 and CPA + BUS 10 groups had significantly higher (*p* < 0.001) α-klotho levels compared to the CPA group. Furthermore, in comparison to CPA + BUS 5, the α-klotho serum levels in the CPA + BUS 10 group were significantly higher (p < 0.01), as presented in Fig. 4.

### BUS stimulated hepatic Nrf2 and HO-1 expression

**Fig. 5:**
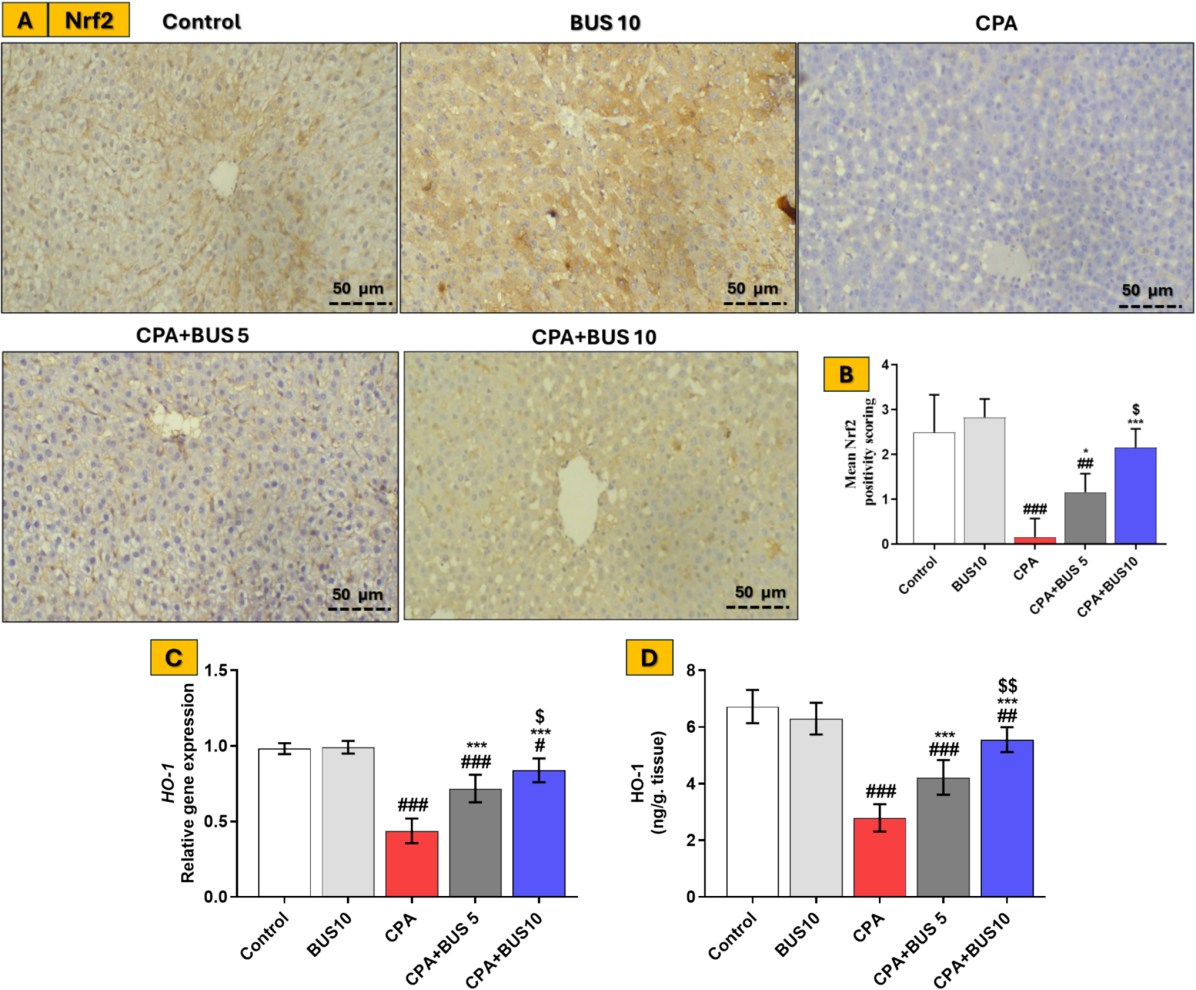
Influence of BUS on Nrf2/HO-1. Images showing the effects of BUS on Nrf2 expression in the liver (**A**) and mean Nrf2 positive scores, *n* = 3 (**B**), among different groups of animals (× 200). Hepatic HO-1 gene expression evaluated by RT-qPCR, *n* = 6 (**C**), and Hepatic HO-1 protein expression evaluated by ELISA, *n* = 6 (**D**). The results are expressed using the mean ± SD. The significance of differences was evaluated using the Kruskal–Wallis test, followed by Dunn’s multiple comparison test for Nrf2 positive scores, while the significance of differences was evaluated using one-way ANOVA, followed by the Tukey–Kramer post hoc test for HO-1 expressions, where ^**#**^*p* < 0.05, relative to the control group, ^**##**^*p* < 0.01, relative to the control group, ^**###**^*p* < 0.001, relative to the control group, *p < 0.05, relative to the CPA group, ***p < 0.001, relative to the CPA group, ^$^*p* < 0.05, relative to the CPA + BUS 5 group, and.^$$^*p* < 0.01, relative to the CPA + BUS 5 group. BUS: buspirone, CPA: cyclophosphamide, Nrf2: Nuclear factor erythroid 2-related factor 2, and HO-1: Heme oxygenase 1

We utilized immunohistochemistry to measure the activity of Nrf2 and RT-qPCR to assess the gene expression of its downstream effector, HO-1. The protein expression of HO-1 was confirmed by ELISA. As shown in Fig. 5, CPA demonstrated a significant decrease (*p* < 0.001) in the immune expression of Nrf2 and both gene and protein expression of HO-1 when compared to the control group. Pretreatment with BUS significantly increased (*p* < 0.001) the hepatic expression of Nrf2 and the expression of HO-1 in a dose-dependent manner compared to the CPA-intoxicated group, indicating that BUS can upregulate the Nrf2/HO-1 cascade to elicit a mitigating effect against CPA-induced hepatotoxicity.

### BUS downregulated hepatic apoptosis

**Fig. 6:**
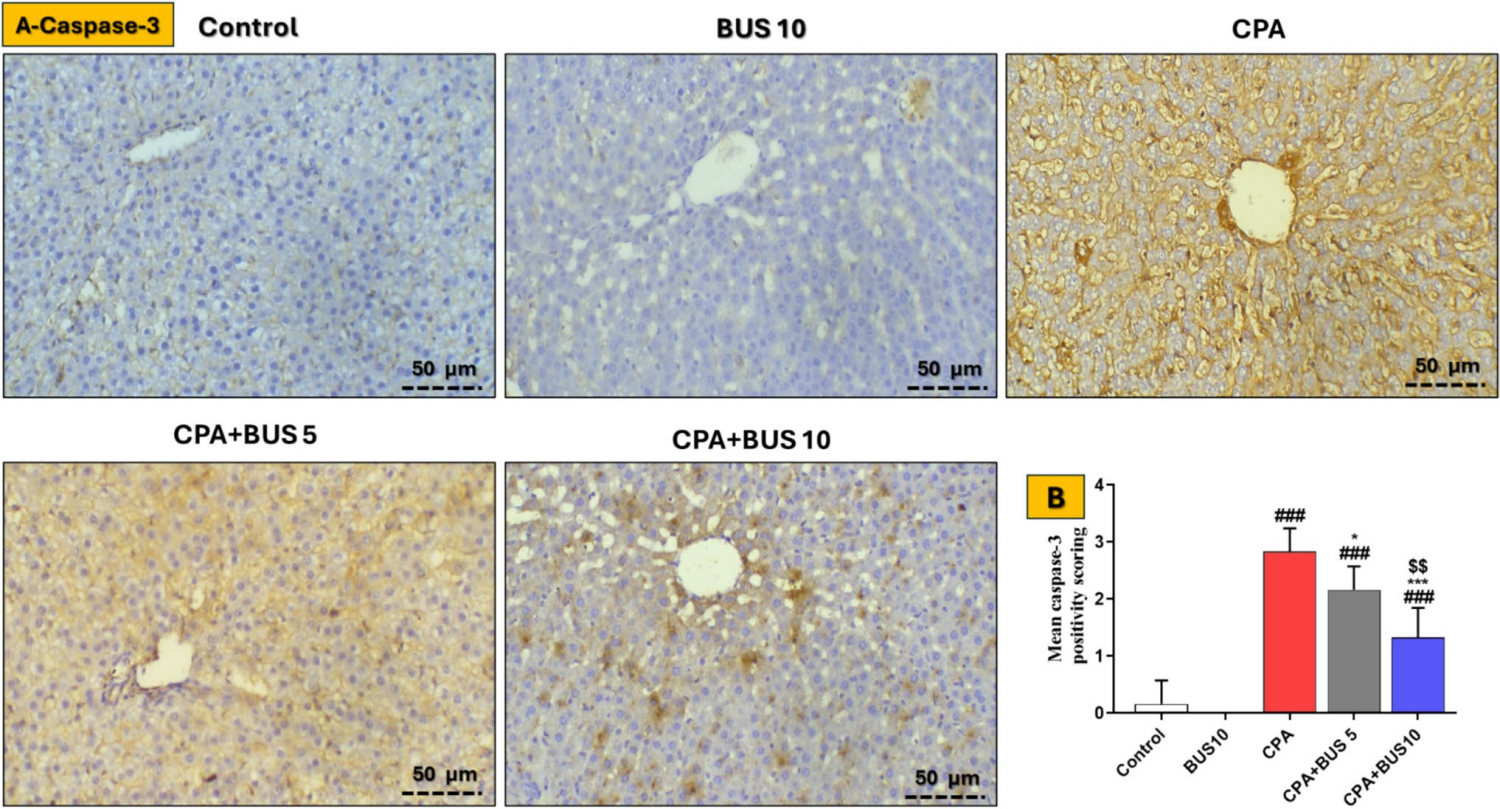
Influence of BUS on caspase-3 expression. Images showing the effects of BUS on caspase-3 expression in the liver (**A**) and mean caspase-3 positive scores (**B**) among different groups of animals (× 200). The mean ± SD is utilized to express the data (*n* = 3). The significance of differences was evaluated using the Kruskal–Wallis test, followed by Dunn’s multiple comparison test, where ^**###**^*p* < 0.001, relative to the control group, *p < 0.05, relative to the CPA group, ***p < 0.001, relative to the CPA group, and ^$$^*p* < 0.01, relative to the CPA + BUS 5 group. BUS: buspirone, and CPA: cyclophosphamide

We detected caspase-3 expression using immunohistochemistry. Figure 6 shows that CPA significantly raised (*p* < 0.001) caspase-3 immune expression compared to the control group. The BUS-treated groups showed a dose-dependent reduction (*p* < 0.001) in hepatic caspase-3 expression comparable to the CPA group.

### BUS minimized histological abnormalities caused by CPA

**Fig. 7:**
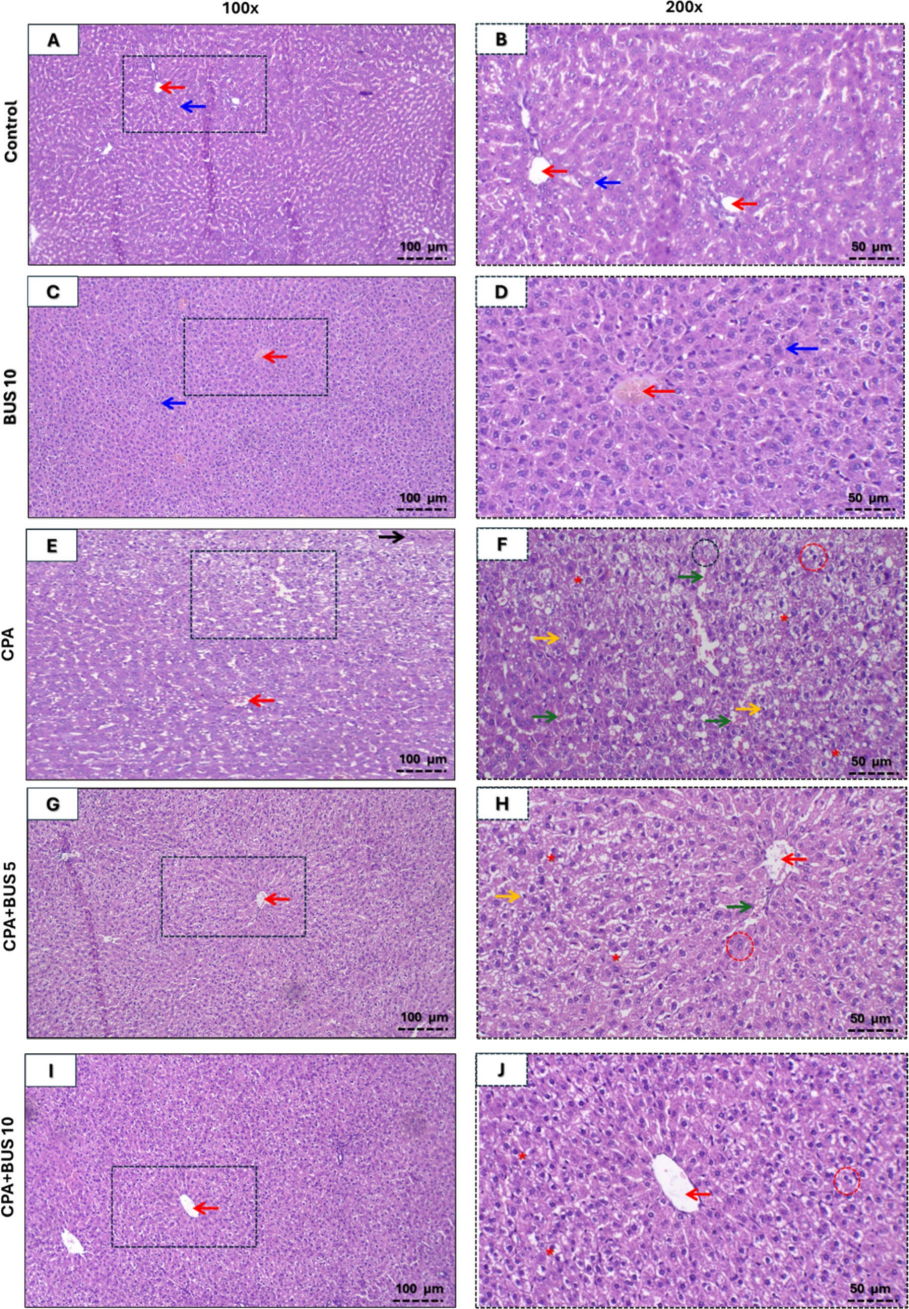
Images illustrating the influence of BUS on the hepatic tissue from different animal groups (*n* = 6) (× 100, × 200). Central vein (red arrow), hepatocytes (blue arrow), enlarged congested sinusoids (green arrow), apoptosis (yellow arrow), inflammation (black arrow), necrosis (black circle), diplocytes (red circle), and steatosis (red star) are all depicted in this diagram

No abnormalities were detected according to any of the parameters of analysis when the histological examination of liver samples from the control and BUS monotherapy groups was performed (Fig. 7A–D). On the other hand, CPA-treated liver tissue showed moderate inflammation and necrosis in addition to prominent steatosis, severe dilated and congested sinusoids and central vein, and many diplocytes (Fig. 7E–F). Treatment with BUS markedly attenuated all studied parameters in a dose-dependent manner relative to the CPA group. Figure 7G–H further illustrates the moderate sinusoidal congestion, steatosis, and apoptosis, along with mild central vein congestion, necrosis, inflammation, and diplocytes in the CPA + BUS 5 group. The BUS 10-treated group exhibited minor evidence of apoptosis, congested central vein, diplocytes, and steatosis, without any patchy necrosis or sinusoidal congestion, as seen in Fig. 7I–J. The scoring for the elements of liver injury is illustrated in Table 4.

**Table 4:** Scoring for the elements of liver injury among groups

Groups	Control	BUS 10	CPA	CPA + BUS 5	CPA + BUS 10
Necrosis	0	0	2	1	0
Apoptosis	0	0	3	2	1
Inflammation	0	0	2	1	0
Central vein congestion	0	0	2	1	1
Sinusoids congestion	0	0	3	2	0
Steatosis	0	0	3	2	2
Diplocyte	0	0	3	1	1
Total scoring	0	0	18^**###**^	^###, *^10	5^##, ***, $$^

## Discussion

Recent data demonstrated that the increased risk of hepatotoxicity associated with CPA restricts the drug’s extensive therapeutic uses (Zhu et al. 2015; Ming et al. 2019; Subramaniam et al. 2013). Hepatic cells are particularly vulnerable to the toxic effects of CPA metabolites because metabolic activation of CPA is required (Saleh et al. 2024). Thus, it is crucial to find innovative and efficient hepatoprotective drugs to improve its therapeutic efficacy. Previous experimental investigations have demonstrated that BUS has the capacity to mitigate hepatic injury induced by carbon tetrachloride and methylphenidate. (Abdel-Salam et al. 2017; Alam And Ikram 2018). In light of these considerations, we postulated that BUS may serve as a viable therapeutic agent to lessen the severity of liver damage induced by CPA.

A significant rise in serum levels of direct bilirubin, hepatic enzymes ALT and AST, and a noticeable decrease in hepatic function were seen after CPA administration. The results are consistent with previous research that found elevated levels of these enzymes in the blood after CPA (El-Beheiry et al. 2023; Lixin et al. 2019; Abdelfattah-Hassan et al. 2019). It is interesting to note that BUS pretreatment caused a dose-dependent, notable decrease in liver damage indicators, including ALT, AST, and direct bilirubin. Our findings align with earlier reports that have demonstrated comparable hepatoprotective benefits of BUS (Abdel-Salam et al. 2017; Alam And Ikram 2018).

It is widely acknowledged that oxidative stress (OS) is a key component leading to liver damage, and it is caused by the formation of harmful metabolites and free radicals during the metabolism of CPA (Zhu et al. 2015; Lixin et al. 2019; Aladaileh et al. 2019). Overproduction of MDA and a reduction in hepatic GSH levels are caused by a chain reaction that begins with acrolein free radicals, which are byproducts of CPA metabolism (Oyagbemi et al. 2016; Jiang et al. 2019). Our results, which demonstrated that CPA-intoxicated rats had higher MDA and lower GSH levels than the control group, were consistent with these earlier findings. Unexpectedly, the present study found that 5 and 10 mg/kg of BUS pretreatment considerably reduced the redox aberrations caused by CPA. Consistent with prior findings demonstrating BUS’s antioxidant action across several experimental investigations, this was demonstrated by an increase in hepatic GSH levels and a decrease in MDA levels (Abdel-Salam et al. 2017; Althagafy et al. 2023; Thomas Broome And Castorina 2022).

Some previous research has linked the activation of NF-κB to increased levels of ROS (Lingappan 2018; Tang et al. 2023). Afterward, the nucleus becomes the home for the activated NF-κB, which then regulates the synthesis of cytokines that promote inflammation, including IL-1β, IL-18, and TNF-α (Khallaf et al. 2023; Baker et al. 2011). Our research shows that an alarming rise in hepatic content of NF-κB and the excessive production of TNF-α, IL-1β, and IL-18 are associated with CPA-evoked liver damage. Multiple studies have shown that CPA-induced inflammation in the liver is orchestrated by these inflammatory mediators (El-Beheiry et al. 2023; Ma et al. 2021; Mansour et al. 2017b). In contrast, pretreatment with BUS at dosages of 5 and 10 mg/kg resulted in a notable decline in hepatic NF-κB, TNF-α, IL-1β, and IL-18. The findings provide light on BUS’s anti-inflammatory capabilities. Our results are in line with previous studies, showing that BUS effectively reduces the overproduction of inflammatory cytokines in a variety of animal models (Althagafy et al. 2023; Sharifi et al. 2015a; Thomas Broome et al. 2021).

Multiple lines of evidence have established that pyroptosis contributes to the development of hepatic damage evoked by CPA (Ma et al. 2021; Mostafa et al. 2022; Mansour et al. 2017a). To verify the role of pyroptosis and to elucidate the mechanisms by which BUS prevented liver damage caused by CPA, we assessed the NF-κB, NLRP3, and caspase-1 protein levels. The NLRP3 is stimulated by ROS either directly or through activated NF-κB. This cleaves pro-caspase 1 into mature caspase 1, which in turn increases the release of mature IL-1β and IL-18 as well as pyroptotic cell death (Mangan et al. 2018). The current results corroborated the previous studies that indicated the highest levels of NF-κB, NLRP3, and caspase-1 expressions in the group that received CPA treatment. In a way that was dependent on the dosage, BUS remarkably reduced the levels of NF-κB, NLRP3, and caspase-1 protein. The results are consistent with those of Althagafy et al. (2023), who postulated that the NLRP3 signaling pathway may be responsible for BUS’s capacity to mitigate methotrexate-induced hippocampal damage (Althagafy et al. 2023).

Additionally, apoptosis is involved in the development of CPA-induced hepatotoxicity, according to many lines of evidence (Hamzeh et al. 2018; Rezaei et al. 2023). CPA’s bioactivation in the liver results in the generation of toxic metabolites such as acrolein and phosphoramide mustard, leading to DNA strand breaks and severe OS (Saleh et al. 2024; Khallaf et al. 2025). The resulting accumulation of ROS and diminished antioxidant defenses directly activate mitochondrial apoptotic pathways (Temel et al. 2020). This is evidenced by increased hepatic caspase-3 activity and the characteristic features of apoptotic cell death in histopathology. Furthermore, upregulation of proinflammatory cytokines (such as TNF-α and IL-1β) and activation of transcription factors like NF-κB amplify apoptotic signaling, while also facilitating crosstalk with pyroptotic pathways via NLRP3 inflammasome and caspase-1 (Sharata et al. 2025b; Alshehri et al. 2025). Examining caspase-3 protein expression allowed us to establish the apoptosis’s role and shed light on how BUS protects against CPA-induced liver damage. This study highlighted the harmful effects of CPA by showing that hepatic caspase-3 levels were significantly higher in the CPA group than in the control group. The surprising finding that BUS effectively prevented hepatic cell death was supported by its dose-dependent reduction of hepatic caspase-3 expression. Consistent with other studies, our results show that BUS has anti-apoptotic effects (Althagafy et al. 2023; Sharifi et al. 2015b).

Additionally, it has been shown that the Nrf2/HO-1 cascade is inhibited by excessive ROS production as an antioxidant defense mechanism (Al-Amarat et al. 2022; Mahmoud et al. 2017). On the other hand, drug-mediated Nrf2 activation dramatically reduced the liver damage brought on by CPA (Mahmoud And Al Dera 2015; Alruhaimi 2023). Our findings indicated that BUS mitigated CPA-induced liver damage by elevating Nrf2 and HO-1 hepatic expression. Similarly, another study found that BUS upregulated Nrf2 and HO-1 expression (Althagafy et al. 2023). Furthermore, research has demonstrated that BUS enhances the Nrf2/HO-1 pathway, which in turn protects the retina and decreases inflammation and OS in age-related macular degeneration (Biswal et al. 2015). Furthermore, the NF-κB/NLRP3/caspase-1 signaling pathway is suppressed by the restoration of Nrf2 activity, which lowers inflammation and pyroptotic cell death (El-Agamy et al. 2018; Hou et al. 2018).

Circulating α-klotho is linked to liver damage and functions as a hormone that reduces oxidative stress (Chi et al. 2023; Kim et al. 2013; Liu et al. 2022). Additionally, it modulates several cascades, including NLRP3-mediated pyroptosis and the Nrf2 signaling cascade (Li et al. 2019; Oh et al. 2015; Xing et al. 2021). In light of these factors, we assessed the α-klotho levels in the serum. The α-klotho level significantly decreased when CPA was given in comparison to the control rats. Fortunately, the anti-aging and antioxidant properties of BUS were demonstrated by the significant increase in circulating α-klotho levels. By modifying many cascades, including Nrf2/HO1 and NF-κB/NLRP3/caspase-1, AMPK can reduce inflammation and OS, according to recent research (Fischhuber et al. 2020; Abd El-Fattah et al. 2022; Bai et al. 2022) Our results showed a significant decrease in hepatic p-AMPK in the CPA-intoxicated group as compared to the control rats. Previous studies that showed p-AMPK downregulation and concurrent OS activation in CPA-induced hepatic damage supported our findings (Bokhary et al. 2022). Conversely, BUS pretreatment increased hepatic p-AMPK to combat OS. These results are consistent with a previous study that demonstrated BUS corrected metabolic imbalances and elevated AMPK expression in a hypertensive rat model (Lee et al. 2023).

These findings may shed light on the histological investigation’s conclusions, which showed that the groups that were pretreated with BUS had liver tissue that was less damaged and had its lobular structure restored, with very little inflammation, steatosis, dilated congested sinusoids, apoptosis, necrosis, and a lower associated scoring grade. These findings match a previous study that established the hepatoprotective advantages offered by BUS (Abdel-Salam et al. 2017).

Several lines of evidence suggest that CPA-induced liver injury is the cumulative outcome of interconnected molecular processes, notably oxidative stress, inflammation, pyroptosis, and apoptosis (Sharata et al. 2025a; Hamzeh et al. 2018). The initial burst of ROS following hepatotoxic insult leads to glutathione depletion and lipid peroxidation, as reflected by altered GSH and MDA levels in our model. This oxidative environment activates NF-κB signaling, enhancing the transcription of proinflammatory cytokines (TNF-α, IL-1β, IL-18) that both perpetuate liver inflammation and promote the assembly of inflammasome complexes like NLRP3 (Srirangan et al. 2025; Abdelnaser et al. 2025a). Activated NLRP3 inflammasome further drives caspase-1-mediated pyroptosis, characterized by membrane rupture and release of inflammatory mediators and intracellular contents (Ma et al. 2021; Sharata et al. 2025c). Simultaneously, oxidative stress and inflammatory cascades converge on apoptotic machinery, elevating caspase-3 levels and facilitating DNA fragmentation and programmed cell death (Mohyeldin et al. 2025; Abdelnaser et al. 2025b). Crosstalk exists whereby cytokine and pyroptosis signaling can amplify apoptotic cell loss, and vice versa, forming a self-propagating cycle of tissue damage. Thus, the mitigation of oxidative, inflammatory, apoptotic, and pyroptotic markers observed with buspirone treatment in this study likely reflects the drug’s ability to interrupt this harmful cascade, ultimately protecting hepatocytes from diverse cell death pathways.

This study provides significant insights into the protective effects of BUS against CPA-induced liver injury with the involvement of key molecular pathways. However, definitive mechanistic causality remains to be established. Future investigations employing pathway-specific inhibitors or activators (e.g., dorsomorphin for AMPK, ML385 for Nrf2) are warranted to validate the role of these pathways. Additionally, a major limitation is the absence of evaluation regarding potential interactions between BUS and the antitumor efficacy of CPA, which should be addressed in tumor-bearing models to determine whether BUS co-administration affects CPA’s cytotoxic activity or tumor progression metrics. Expanding the panel of hepatic function biomarkers to include GGT and alkaline phosphatase will also enhance the comprehensiveness of liver injury assessment in future research. Ultimately, clinical trials examining various dosing regimens and treatment durations of BUS are necessary to translate these preclinical findings into effective therapeutic strategies for mitigating chemotherapy-induced hepatotoxicity.

**Fig. 8:**
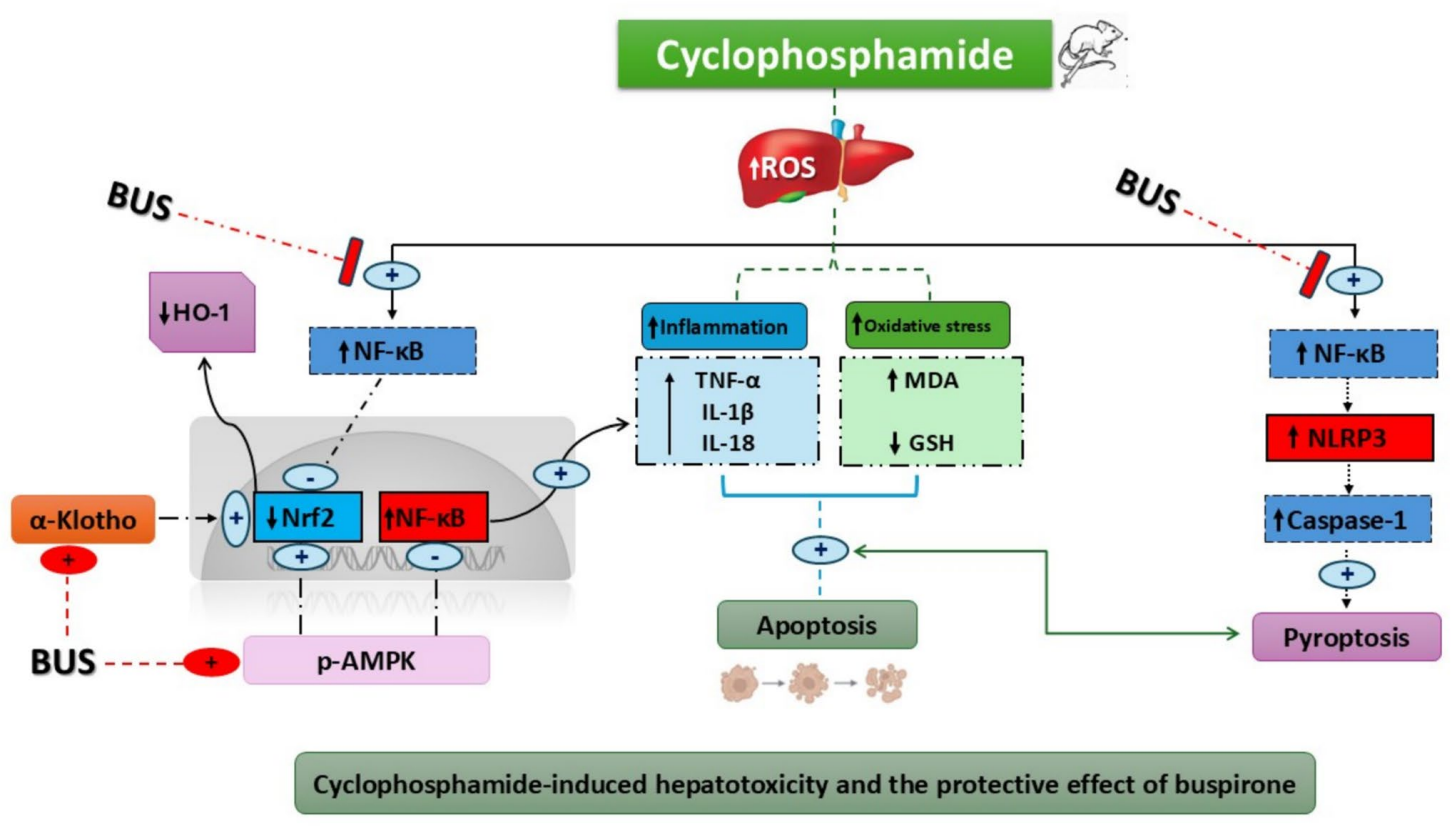
A graphic overview illustrates the hepatotoxic effects of CPA and the suggested mechanism of BUS. ROS: reactive oxygen species, NF-κB: nuclear factor-kappa B, Nrf2: Nuclear factor erythroid 2-related factor 2, HO-1: Heme oxygenase 1, p-AMPK: phosphorylated adenosine monophosphate-activated protein kinase, TNF-α: tumor necrosis factor-alpha, IL-1β: interleukin-1β, IL-18: interleukin-18, NLRP3: NOD-like receptor family pyrin domain containing 3, MDA: malondialdehyde, GSH: reduced glutathione, and BUS: buspirone

Finally, the current study demonstrated that BUS markedly attenuated OS and pyroptosis in CPA-induced hepatic injury in rats. The hepatoprotective effects of BUS are closely linked to its ability to inhibit the NF-κB/NLRP3 inflammasome axis, thereby reducing inflammation and OS, while simultaneously activating the AMPK/Nrf2/HO-1 pathway. In addition, our findings highlighted a pivotal role for α-klotho activation in mediating these protective mechanisms, as illustrated in Fig. 8.

## Conclusion

In the end, this study provides the first concrete evidence of BUS’s critical function in reducing CPA-induced liver damage. Thus, in addition to BUS’s advantages for alleviating dyspnea, emesis, and gastroparesis, which justify its use in cancer patients, BUS may have a promising therapeutic potential for improving CPA’s tolerability by reducing its undesirable consequences, particularly hepatotoxicity.

## Supplementary Information

Below is the link to the electronic supplementary material.

ESM1 (PDF 330 KB)

ESM2 (PDF 342 KB)

## Data Availability

All data produced or examined during this investigation are incorporated in this published article.
