# Supplementary material for: Buspirone combats cyclophosphamide-provoked hepatotoxicity in rats via activation of AMPK/Nrf2/HO-1 and suppression of NF-κB p65 /NLRP3 inflammasome pathways
Source: Naunyn Schmiedebergs Arch Pharmacol. 2025 Nov 3;399(4):5483–98. doi: 10.1007/s00210-025-04718-3 (PMC13046598; doi:10.1007/s00210-025-04718-3)

Repeat 1

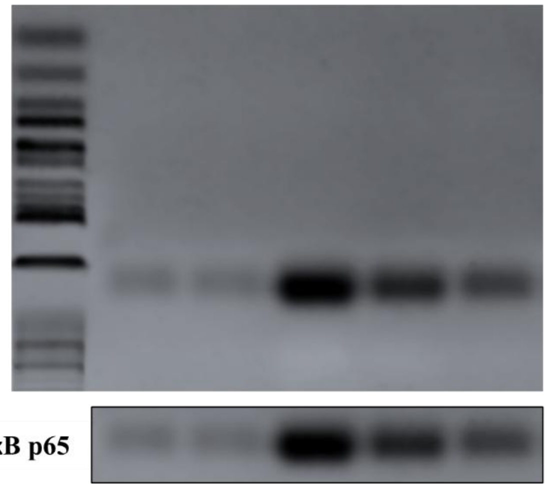

Repeat 2

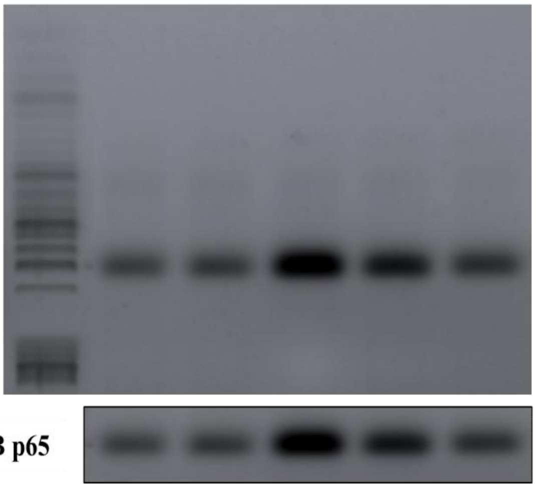

Repeat 3

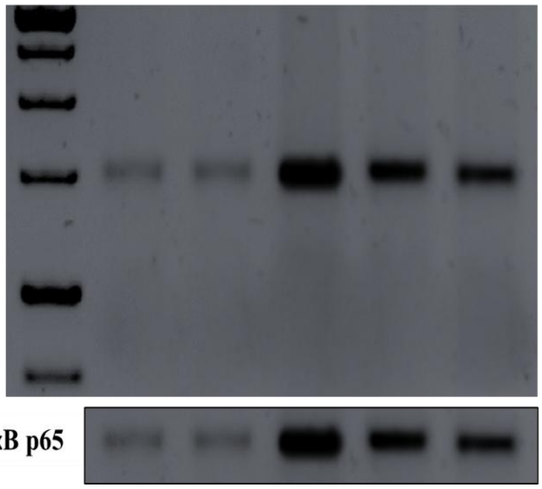

Repeat 1

NLRP3

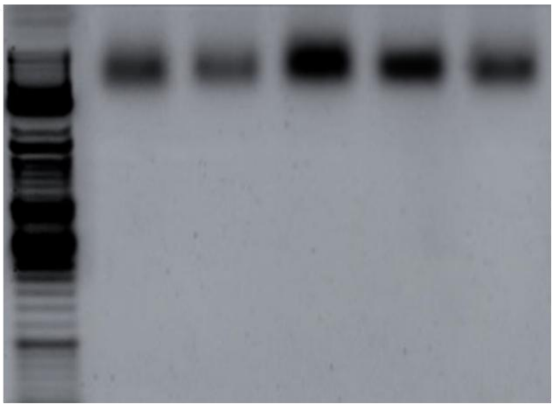

NLRP3

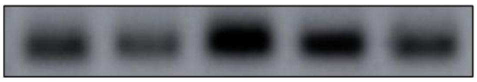

118 KDa

Repeat 2

NLRP3

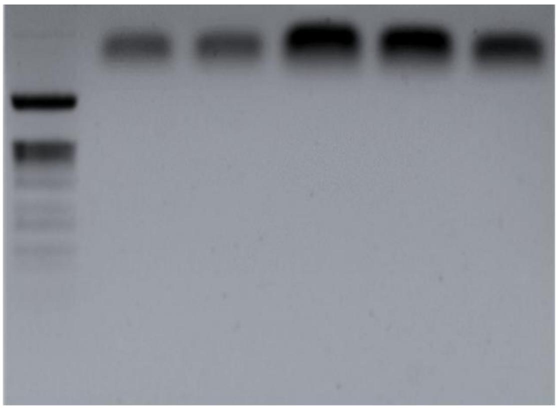

NLRP3

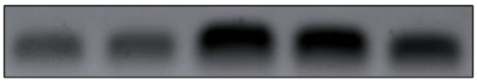

118 KDa

Repeat 3

NLRP3

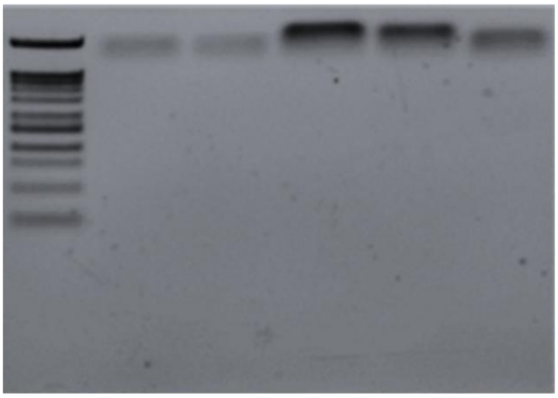

NLRP3

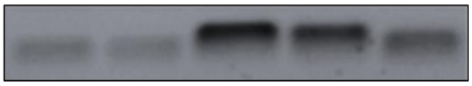

118 KDa

Repeat 1

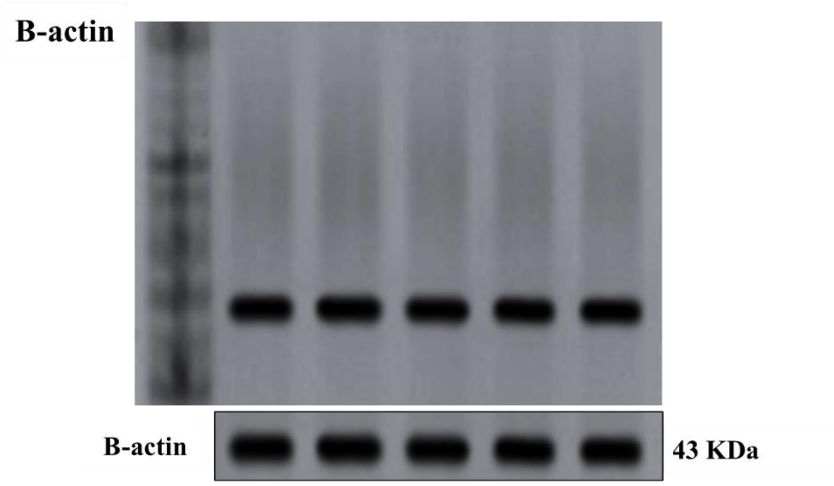

Repeat 2

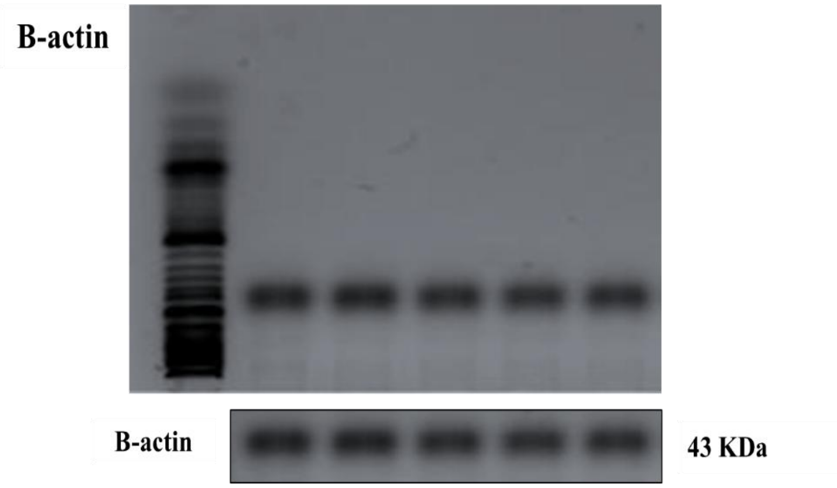

Repeat 3

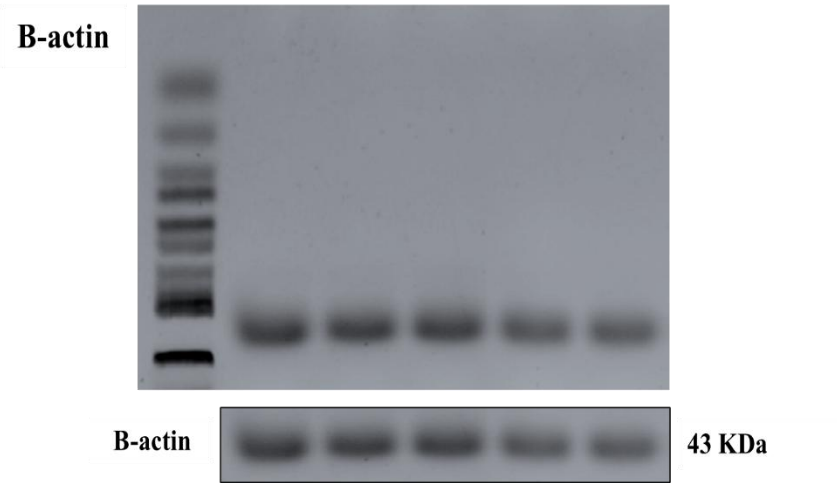

Supplement: Supplementary file 1 — (PDF 330 KB) [file 210_2025_4718_MOESM1_ESM.pdf]
