# Supplementary material for: Buspirone combats cyclophosphamide-provoked hepatotoxicity in rats via activation of AMPK/Nrf2/HO-1 and suppression of NF-κB p65 /NLRP3 inflammasome pathways
Source: Naunyn Schmiedebergs Arch Pharmacol. 2025 Nov 3;399(4):5483–98. doi: 10.1007/s00210-025-04718-3 (PMC13046598; doi:10.1007/s00210-025-04718-3)
Supplement: Supplementary file 2 — (PDF 342 KB) [file 210_2025_4718_MOESM2_ESM.pdf]

Repeat-1

p-AMPK

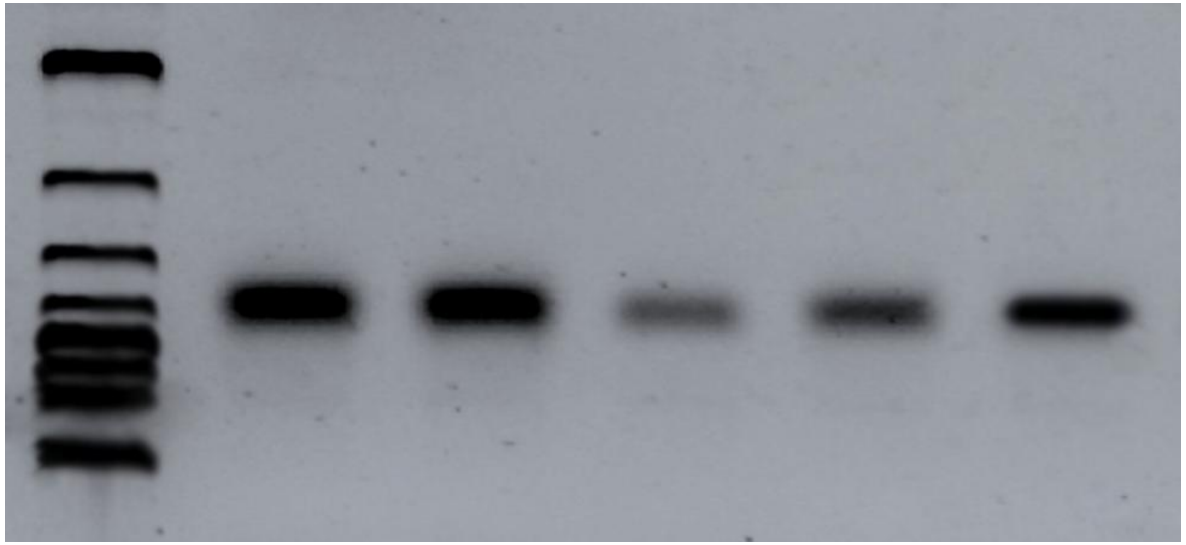

p-AMPK

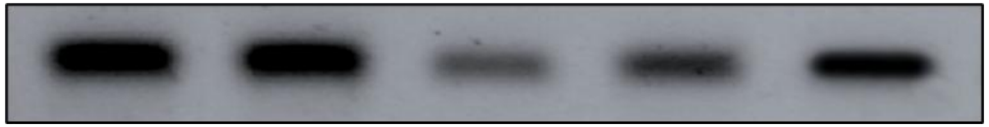

62 KDa

Repeat-1

AMPK

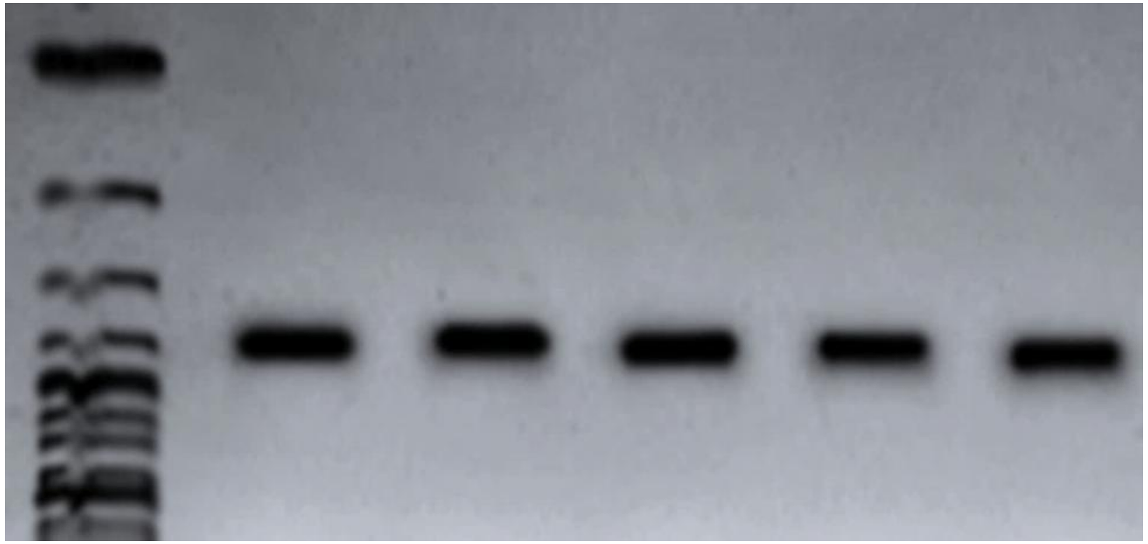

AMPK

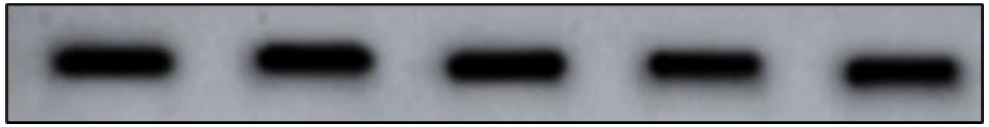

62 KDa

Repeat-2

p-AMPK

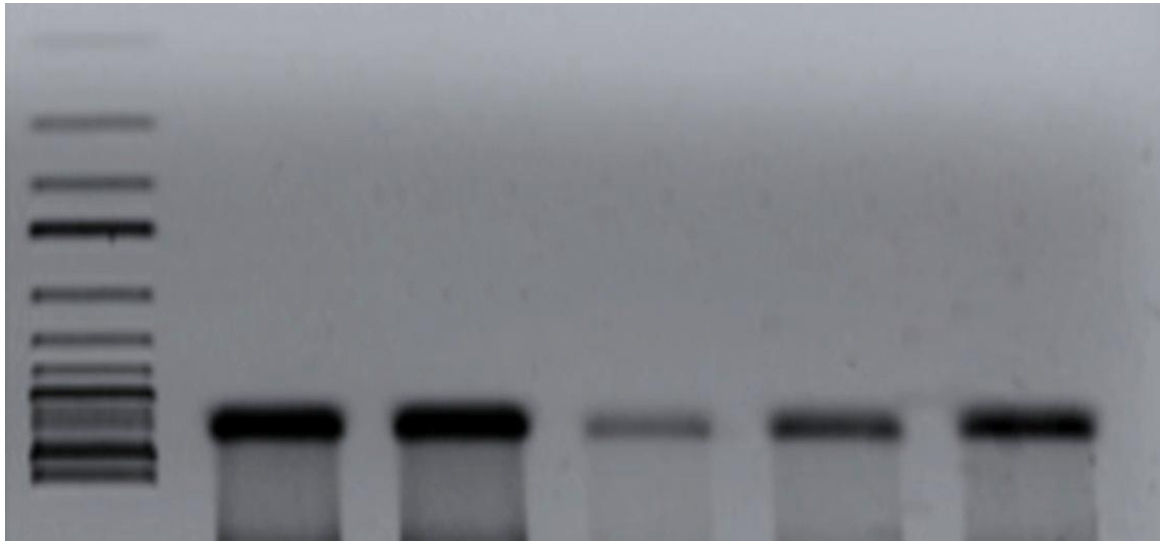

p-AMPK

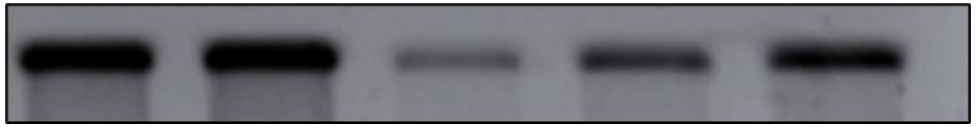

62 KDa

Repeat-2

AMPK

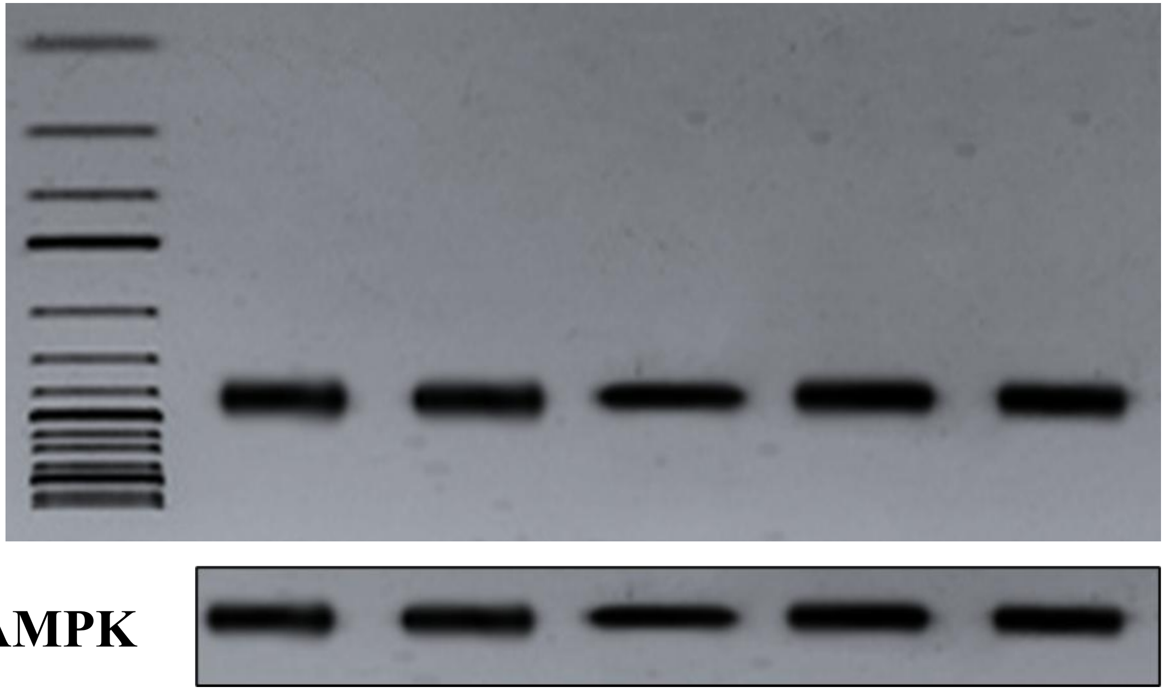

AMPK 62 KDa

Repeat-3

p-AMPK

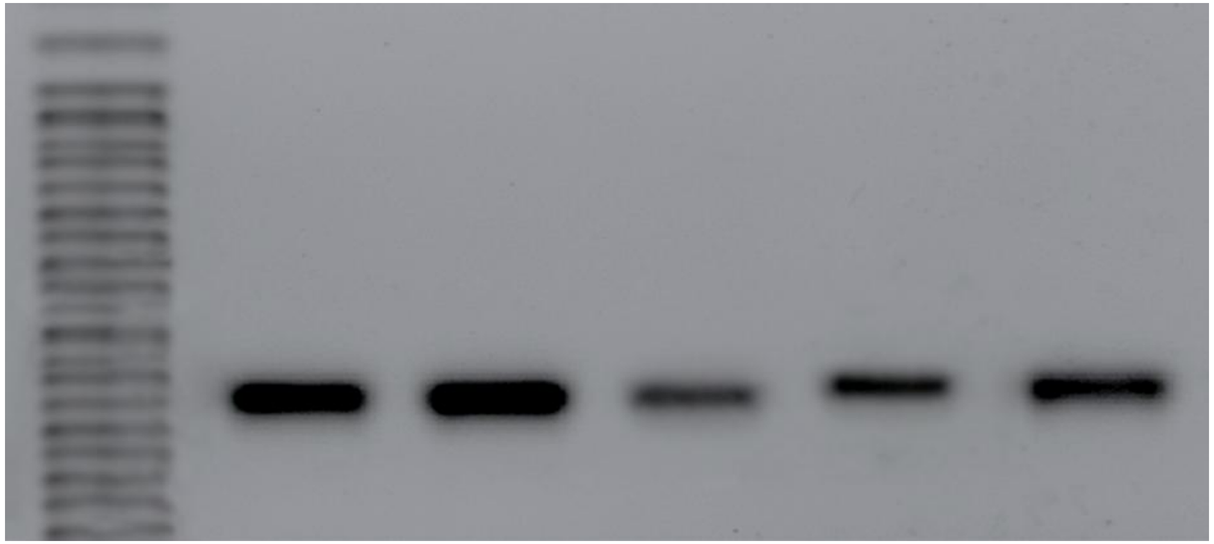

p-AMPK

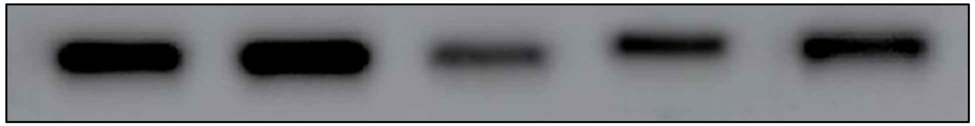

62 KDa

Repeat-3

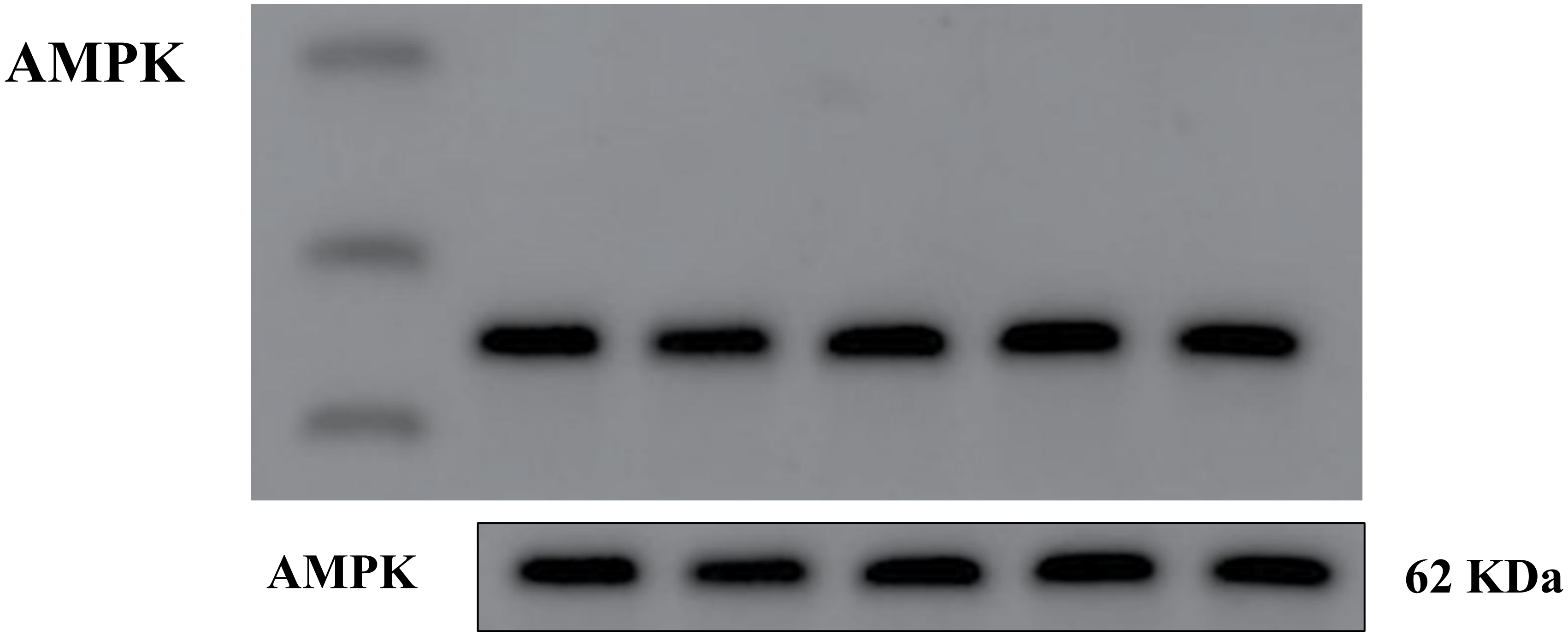

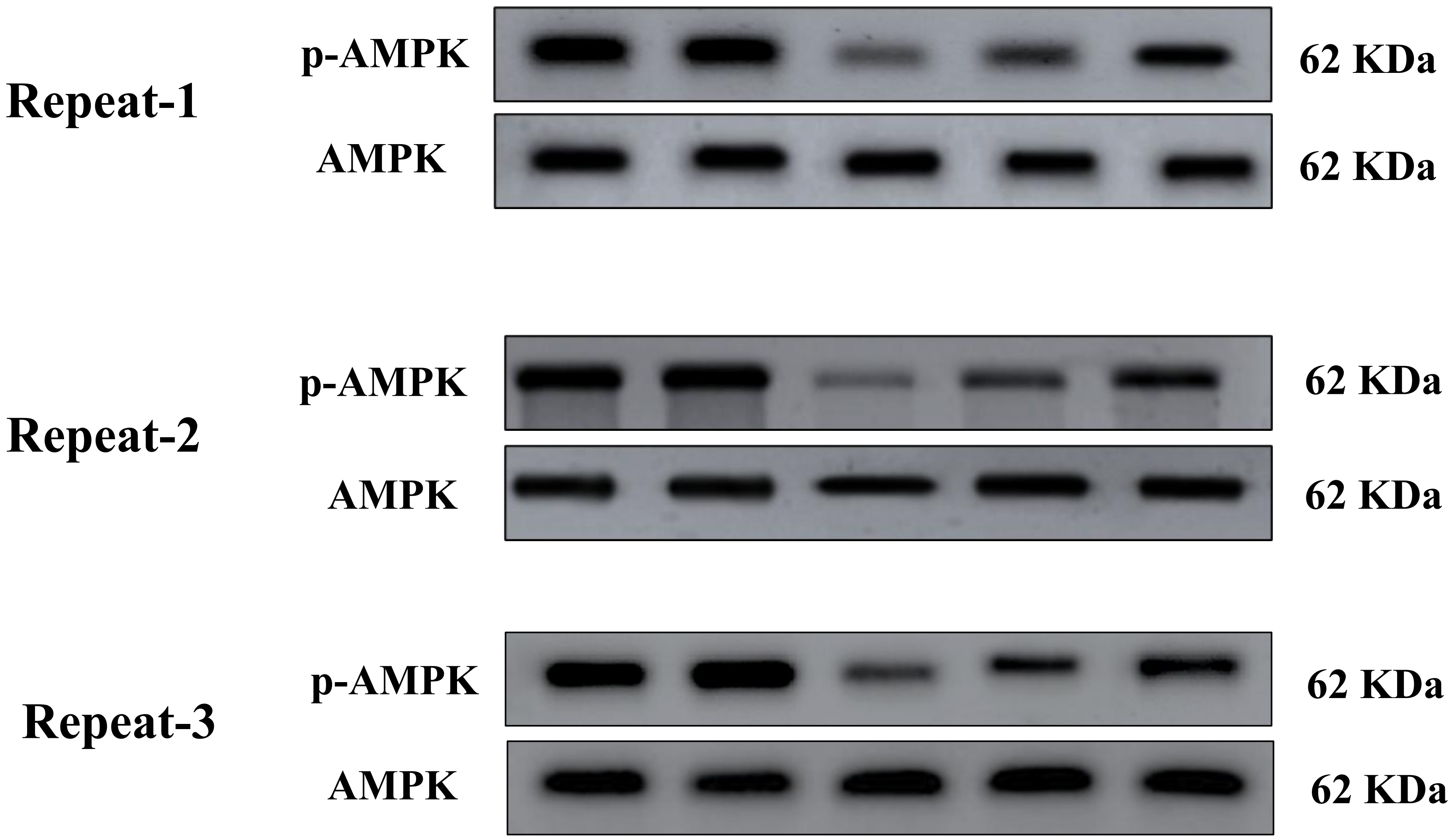
Repeat-1
p-AMPK
62 KDa
AMPK
62 KDa
Repeat-2
p-AMPK
62 KDa
AMPK
62 KDa
Repeat-3
p-AMPK
62 KDa
AMPK
62 KDa

AMPK ELISA

Hepatic p-AMPK was measured using an ELISA kit (ER0730, Fine Biotech Co., China).

BUS enhanced hepatic p-AMPK

The Figure shows that the CPA group had a significant decrease ($p < 0.001$) in hepatic p-AMPK, as compared to the control group. Hepatic p-AMPK levels showed a significant increase ($p < 0.05$, $p < 0.001$, respectively) in the CPA+BUS 5 and CPA+BUS 10 groups compared to the CPA group. The p-AMPK level was also noticeably greater ($p < 0.05$) in the CPA+BUS 10 group compared to the CPA+BUS 5 group.

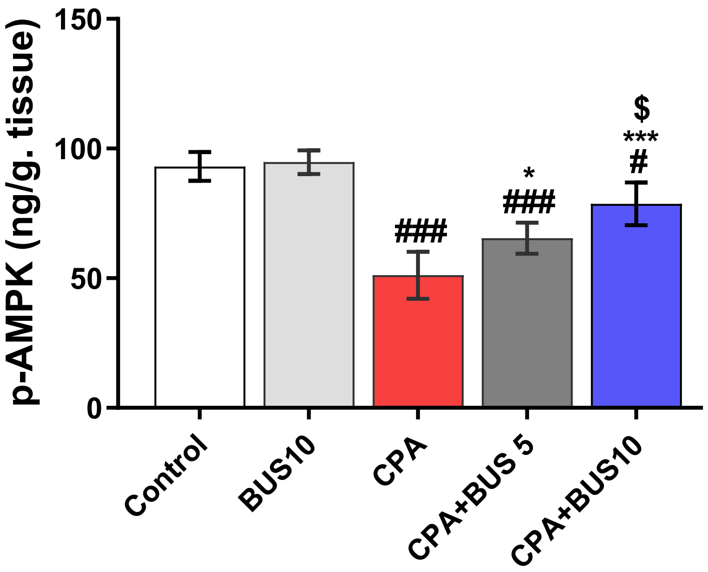

Impact of BUS on hepatic p-AMPK. The results are expressed using the mean ± SD (n = 6). The significance of differences was evaluated using one-way ANOVA, followed by the Tukey-Kramer post-hoc test, where #; *p<0.05*, relative to the control group, ###; *p<0.001*, relative to the control group, *; *p<0.05,* relative to the CPA group, ***; *p<0.001,* relative to the CPA group, and $; *p<0.05,* relative to the CPA+BUS 5 group. BUS: buspirone, CPA: cyclophosphamide, and p-AMPK: Phosphorylated Adenosine monophosphate-activated protein kinase.
